# The Piancatelli Rearrangement: New Applications for an Intriguing Reaction

**DOI:** 10.3390/molecules181012290

**Published:** 2013-10-08

**Authors:** Claudia Piutti, Francesca Quartieri

**Affiliations:** 1 Department of Medicinal Chemistry, Nerviano Medical Sciences srl, Business Unit Oncology, Viale Pasteur 10, 20014 Nerviano, MI, Italy; 2 Department of Chemical Core Technologies, Nerviano Medical Sciences srl, Business Unit Oncology, Viale Pasteur 10, 20014 Nerviano, MI, Italy; E-Mail: francesca.quartieri@nervianoms.com

**Keywords:** 2-furylcarbinols, pentadienyl cation, prostaglandins, spirocycles, domino reaction

## Abstract

Nearly forty years ago, at the University of Rome, Giovanni Piancatelli and co-workers discovered the acid-catalyzed water-mediated rearrangement of 2-furylcarbinols into 4-hydroxycyclopentenones. These motifs are core components of several pharmacologically active compounds and precursors of many natural products. The main features of this reaction are the simple experimental conditions, the stereochemical outcome and the generality of the procedure. Consequently, a re-emergence of this reaction has been seen recently, including developments of the Piancatelli rearrangement with some interesting inter- and intramolecular variants. This review will mainly focus on the general aspects of the reaction along with its more recent applications.

## 1. Introduction

**Scheme 1:**
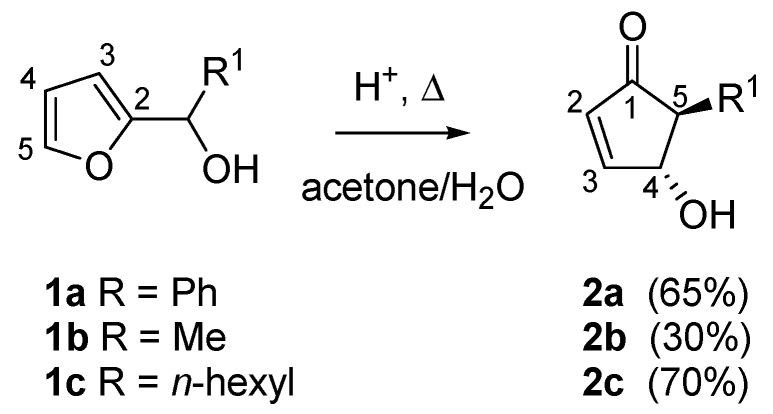
The Piancatelli rearrangement.

In 1976, while studying the reactivity of heterocyclic steroids, Piancatelli and co-workers observed for the first time the rearrangement of a 2-furylcarbinol into a 4-hydroxycyclopent-2-enone in an acidic aqueous system. Following the original report on this transformation [1], the same group continued to systematically investigate the rearrangement [2]. In summary, the heating of 2-furylcarbinols (compounds **1a**–**c**) in an acetone-water solvent system in the presence of strong acids (e.g., formic, polyphosphoric or *p*-toluenesulfonic acid) led to the formation of 4-hydroxy-5-substituted- cyclopent-2-enones (compounds **2a**–**c**), useful intermediates for the synthesis of natural products (Scheme 1).

**Scheme 2:**
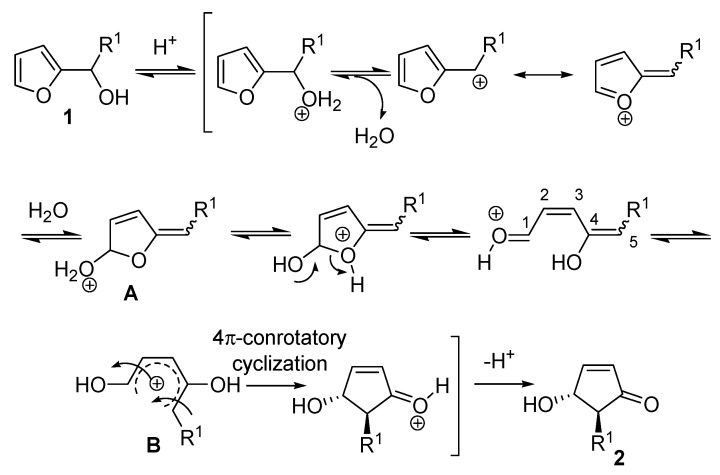
Proposed mechanism of the rearrangement.

The high level of stereochemical control inherent in the rearrangement delivered exclusively the *trans* isomer, as demonstrated by the ^1^H-NMR coupling constant between the two vicinal hydrogens (*J_trans_* = 2.5 Hz). The proposed mechanism involves the formation of a carbocation driven by a protonation-dehydration sequence of the 2-furylcarbinol, the nucleophilic attack of a water molecule then generates intermediate **A** which undergoes ring opening (Scheme 2). The resulting 1,4-dihydroxypentadienyl cation **B**, that adopts a conformation in which the two hydroxy groups are *anti*, provides the *trans*-4-hydroxy-5-substituted-cyclopent-2-enone (**2**) as a racemate, through a 4π-conrotatory cyclization [2].

**Scheme 3:**
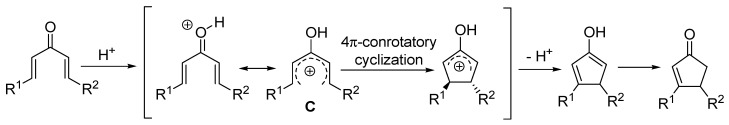
Nazarov cyclization mechanism.

The mechanism shown in Scheme 2 resembles the Nazarov cyclization, in which a divinylketone, activated by Brønsted or Lewis acids, rearranges to a cyclopent-2-enone progressing through a pentadienylic cation (**C**) and a conrotatory ring-closure (Scheme 3) [3,4,5].

De Lera and co-workers supported the pericyclic nature of this rearrangement with theoretical calculations of the energy content of the possible isomeric 1,4-dihydroxypentadienyl cations involved in the mechanism [6]. Furthermore, they attributed the high *trans* stereoselectivity of the reaction to a preferred *out*,*out*-geometry of the cationic intermediate (**B**).

According to the authors’ hypothesis, the enolic hydroxy group at C4 can isomerize to the *outwards* orientation, while the one at C1 should derive from a stereoselective furan ring opening that provides the less congested *out,out*-isomeric form, thus disfavoring isomer with the hydroxy group with an *inwards* position [6].

**Scheme 4:**
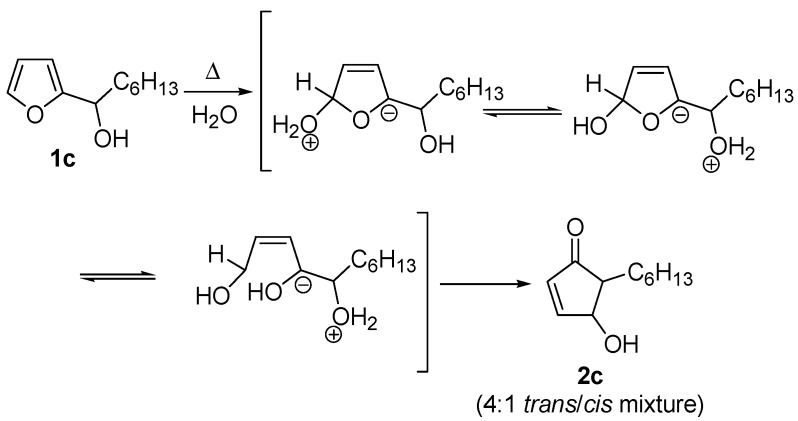
Proposed zwitterion-mediated mechanism.

Despite the electrocyclic process is the most widely accepted, two other possible mechanisms have been proposed for the rearrangement. The first one was described only by D’Auria [7], who invoked zwitterionic intermediates in an attempt to rationalize the formation of the *cis* isomer, along with the more abundant *trans* one, when performing the rearrangement on **1c** (R = small alkyl groups) in boiling water without any acid catalysis (Scheme 4)*.*

**Scheme 5:**
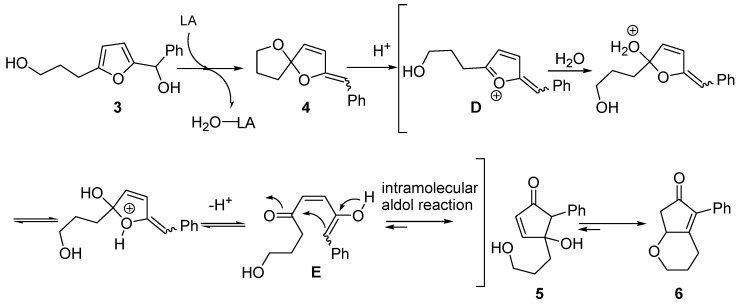
Proposed aldol-type mechanism.

The second alternative mechanism (Scheme 5) was proposed by Yin and co-workers, while investigating the rearrangement of 2-furylcarbinols bearing an hydroxyalkyl chain at the 5 position [8]. For example, conversion of **3** into oxabicyclic cyclopentenone **6**, progressing through intermediate spiroketal enol ether **4** [9,10], was rationalized by envisioning an aldol-type intramolecular addition. Thus, **4** generates intermediate (**D**) in the presence of a Brønsted or Lewis acid. Upon addition of water and prototropic shift, **D** evolves into keto-enol **E** that finally undergoes an intramolecular aldol reaction, leading to intermediate 4-hydroxy-5-substituted-cyclopent-2-enone (**5**). This in turn delivers the more stable derivative **6** [8,11].

The scope and limitations of the Piancatelli rearrangement were previously reviewed in 1982 and 1994 [12,13]. In this review the latest and most interesting features of the reaction, including its new applications will be described.

## 2. An Overview of the Piancatelli Rearrangement

Piancatelli observed that more reactive substrates like 5-methyl-2-furylcarbinols required milder conditions to rearrange in order to avoid side-products. In such cases, weak Lewis acids as ZnCl_2_ could drive the reaction to the desired 4-hydroxy-4-methyl-5-substituted-cyclopent-2-enones, although an equimolar ratio of ZnCl_2_ and substrate was required [14]. It was also noted that alkyl groups on the hydroxy-bearing carbon atom render the starting material more stable and less prone to dehydrate, thus resulting in longer reaction time. In addition the corresponding cationic intermediates are more reactive, consequentially leading to lower yields and the formation of side-products [14].

Among the 5-substituted-2-furylcarbinols, only 5-methyl-2-furylcarbinols [14] and 4-bromo-5-phenyl-2-furylcarbinols undergo rearrangement [15]. Moreover, 5-nitro-2-furyl-derivatives result in no reaction even under harsh conditions, while the 5-methoxy and 5-chloro-2-furylcarbinols lead to the formation of 4-ylidenebutenolides [16,17,18].

**Scheme 6:**
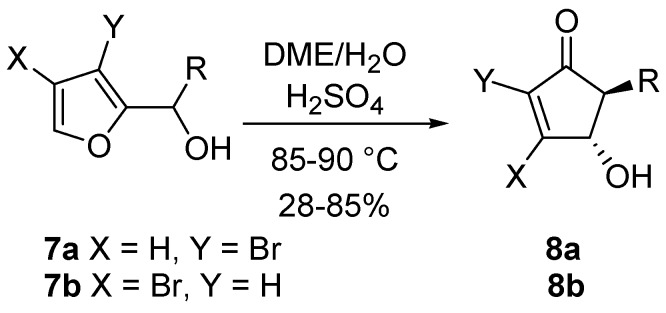
Rearrangement on brominated substrates.

The corresponding 3-bromo (**7a**) and 4-bromo-2-furylcarbinols (**7b**) undergo a stereoselective rearrangement, although under more forcing conditions. The *trans* relationship between the substituents in the resulting cyclopenten-2-ones **8a** and **8b** is however maintained (Scheme 6) [15].

**Scheme 7:**
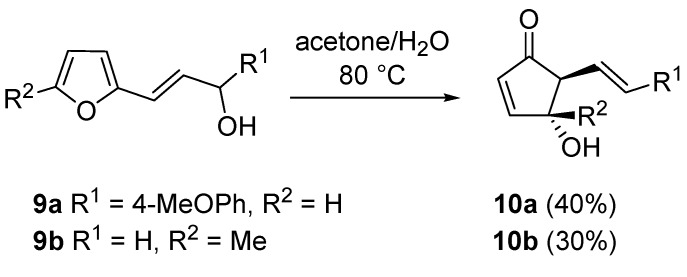
Rearrangement on 2-furyl-alkenyl carbinols.

The same rearrangement was applied to 2-furyl-alkenyl carbinols **9** leading to 5-alkenyl-derivatives **10**, useful intermediates for the synthesis of prostaglandin analogues as the upper side chain at C5 is suitable for further manipulations. Since these compound structures (**9**) are remarkably reactive, a simple solvolysis in an acetone/water mixture was sufficient for the rearrangement to occur (Scheme 7) [19].

**Scheme 8:**
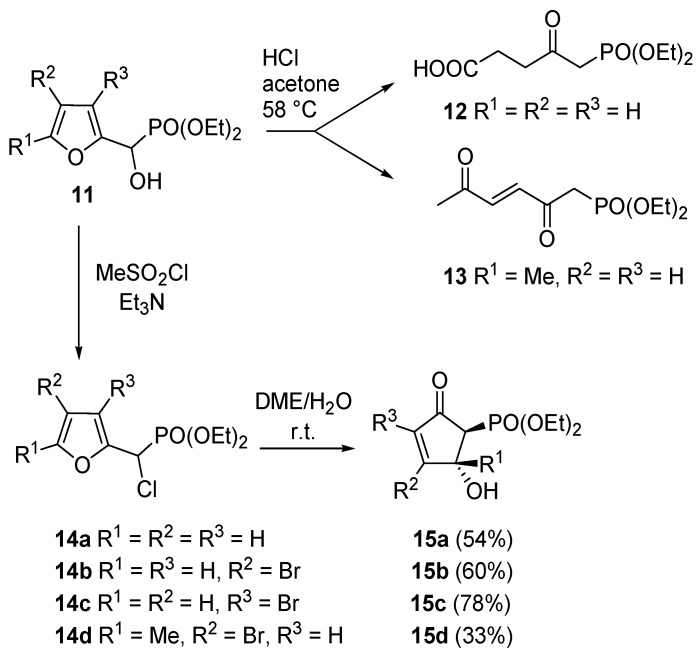
Rearrangement on 2-furyl-hydroxymethylphosphonates.

Piancatelli’s group also investigated the reactivity of 2-furyl-hydroxymethylphosphonates (**11**). In this case however, an acidic treatment of **11** led to levulinic acid derivatives **12** (Marckwald-type products) or to diethyl 2,5-dioxohex-3-enylphosphonate **13** according to the substitution pattern on the furan ring. However, in order to access compounds **15a**–**d**, the hydroxy group had to be converted into a more reactive leaving moiety (e.g., chlorine, **14a**–**d**, Scheme 8) [20].

During the 80s the rearrangement was widely studied by researchers at Sumitomo Chemical Company, Ltd. (Osaka, Japan), in collaboration with Prof. Piancatelli, resulting in several patent applications [21,22,23,24]. After an extensive experimentation, they found that treatment of 2-furylcarbinols (**1**) in an aqueous medium within a specific pH range (3.5–5.8) could afford 4-hydroxycyclopent-2-enones (**2**) in good yields, which also included the normally less reactive substrates (when R was an alkyl, alkenyl and alkynyl group). Furthermore, these experimental conditions increased the reaction rate and minimized the formation of by-products.

Recently, the rearrangement was also studied in a batch reactor under microwave irradiation (300 W), thus dramatically shortening the reaction time (minutes *vs.* several hours) and improving the yields (up to 95%). The scale-up of the rearrangement was optimized by employing a microreactor that allowed the development of a continuous flow process [25].

**Scheme 9:**
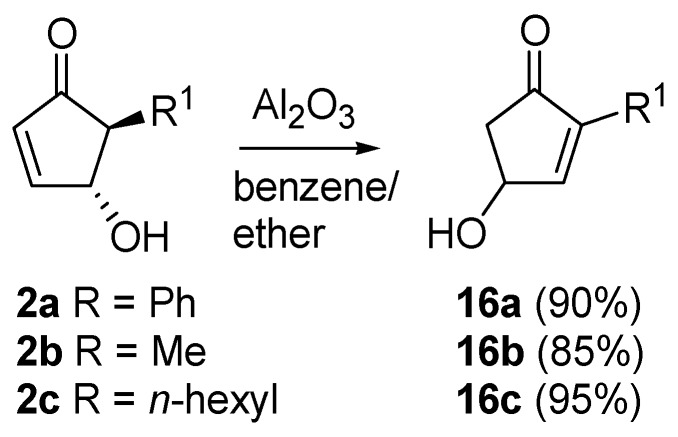
Isomerization to 2-substituted-4-hydroxycyclopent-2-enones.

A very intriguing conversion involving 4-hydroxy-5-substituted-cyclopent-2-enones (**2**) is their isomerization to 4-hydroxy-2-substituted-analogues (**16**), excellent intermediates for prostaglandins synthesis (Scheme 9). This transformation had already been described by Stork *et al.*, who treated **2** with chloral in the presence of triethylamine [26]. Piancatelli *et al.*, also observed this migration during the chromatographic purification of **2** on neutral (for **2a**) or basic alumina (for **2b**–**c**) [27]. The corresponding isomerized products **16** were isolated in high yield.

**Scheme 10:**
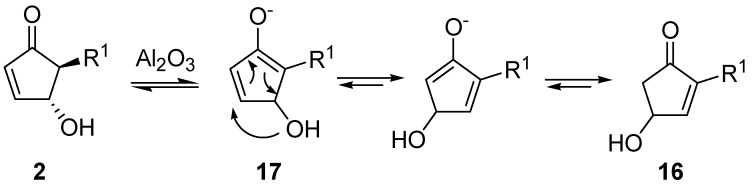
Possible isomerization mechanism.

By extending this isomerization to a series of derivatives and after a careful examination of the mechanism, it was demonstrated that the reaction occurred *via* an intramolecular shift of the hydroxy group on the intermediate enolate **17** (Scheme 10) [28], thus ruling out a dehydration-hydration sequence involving the nucleophilic attack of an external water molecule, as previously supposed [29].

In fact, when adsorbed on methanol-deactivated alumina under anhydrous conditions, **2** delivered isomer **16** quantitatively, with no detectable amounts of the 4-methoxy derivative. On the other hand, 4-acetoxy-5-phenylcyclopent-2-enone isomerizes to 4-acetoxy-2-phenylcyclopent-2-enone after adsorption on neutral alumina and elution with a mixture of benzene and diethyl ether, thus confirming the proposed mechanism. With aromatic R groups, neutral alumina was sufficient for the isomerization to occur, while with aliphatic substituents the employment of basic alumina was necessary to promote the hydroxy shift [28].

**Scheme 11:**
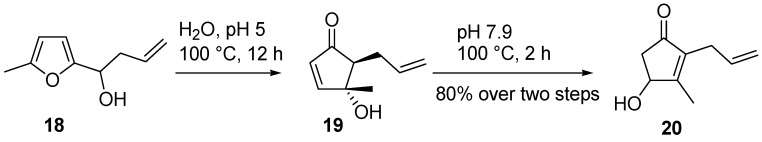
Example of one-pot isomerization.

4-Hydroxy-2-substituted-derivatives **16** can be synthesized in a one-pot procedure from 2-furylcarbinols (**1**) simply by switching from an acidic pH, necessary for the rearrangement, to a basic environment, to promote the isomerization. For example, 5-methyl-2-furylallylcarbinol (**18**) was converted into cyclopentenone **19** in refluxing water over 12 hours at pH 5. Intermediate **19** was not isolated, but it was isomerized to 2-allyl-4-hydroxy-3-methyl-cyclopent-2-enone (**20**) by adjusting the pH to 7.9 and prolonging the reflux for an additional two hours (Scheme 11) [23,24].

The straight transformation of 2-furylcarbinols (**1**) into 4-hydroxy-2-substituted-cyclopent-2-enones (**16**) can also be achieved in a buffered aqueous solution in the presence of MgCl_2_ heated at high temperature (150 °C) in an autoclave, thus allowing to isolate the desired products in high yield (80%–84%) [23,24].

### Applications of the Original Piancatelli Rearrangement

One of the most important applications of the Piancatelli rearrangement is in the synthesis of prostaglandins and their derivatives. Demonstration of the versatility of the domino sequence “2-furylcarbinol rearrangement/isomerization” was shown by Piancatelli himself, who synthesized key intermediates for the preparation of the prostanoic acid skeleton starting from 2-furylcarbinols bearing a second functional group in the side chain [29].

**Figure 1:**
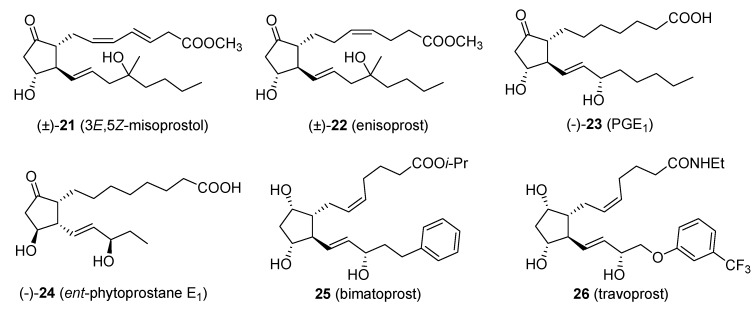
Products obtained *via* the Piancatelli rearrangement.

Some of the products arising from the application of this domino sequence are: 3*E*,5*Z-*misoprostol (**21**) [30], enisoprost (**22**) [31], 4-fluoro-enisoprost [32], 2-normisoprostol [33], prostaglandin E_1_ (**23**) [34], *ent*-phytoprostane E_1_ (**24**) and 16-*epi*-phytoprostane E_1_ [35], bimatoprost (**25**, Lumigan™) and travoprost (**26**, Travatan™) [36,37] (Figure 1).

**Scheme 12:**
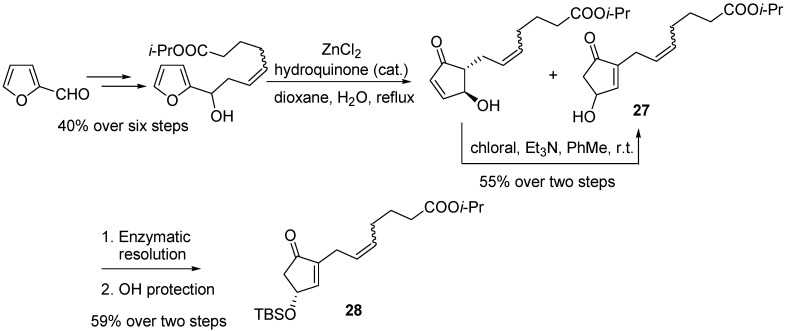
Large scale synthesis of intermediate **28**.

Bimatoprost (**25**) and travoprost (**26**) can be synthesized from the common intermediate 4-silyloxycyclopentenone **28**, whose production was set-up on a kg-scale by exploiting a “Piancatelli rearrangement/chloral-mediated isomerization” sequence, starting from furfural, and subsequent enzymatic resolution of the resulting 4-hydroxycyclopentenone **27** [38] (Scheme 12). 

**Scheme 13:**
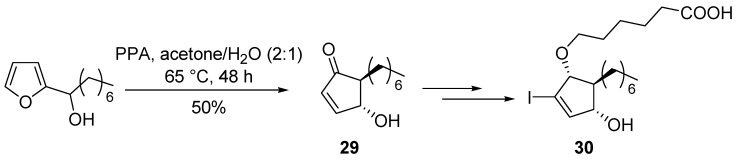
Synthesis of PPARγ agonists.

4-Hydroxycyclopentenones bearing functionalized side chains at the position 2 can also be prepared from *bis*-thioalkylfurans [39]. 5-heptyl-4-hydroxycyclopent-2-enone core structure (**29**) was obtained by Piancatelli rearrangement for the synthesis of selective and potent PPARγ agonists (**30)** (Scheme 13) [40]. 

**Scheme 14:**
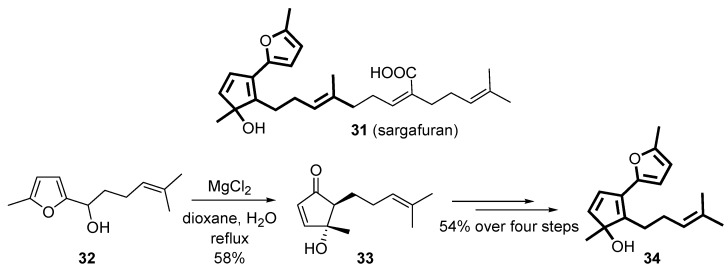
Synthesis of the racemic core framework (**34**) of sargafuran.

The rearrangement of 2-furylcarbinols has also been applied to the total synthesis of natural products [41,42]. Very recently, the core framework of the proposed structure of sargafuran (**31**) was accomplished *via* the Piancatelli rearrangement on intermediate **32** in the presence of MgCl_2_ as the key-step [43]. The use of this Lewis acid allowed to isolate the rearranged product **33** in moderate yield (58%), while other acids (e.g., polyphosphoric acid, ZnCl_2_) led to lower recovery (up to 34%) because of the competitive dehydration of **32** and the formation of unidentified side-products. Subsequent protection of the hydroxy group of intermediate **33**, addition of the furan moiety, dehydration and deprotection led to **34**, a simplified analogue of sargafuran [43] (Scheme 14).

## 3. Recent Versions of the Piancatelli Rearrangement

Several new and efficient applications of this rearrangement have recently appeared in the literature utilizing alternative nucleophiles to water, and the synthetic utility of this reaction has been widely developed. Pharmacologically valuable scaffolds, which were usually prepared via multi-step sequences, can be synthesized in very mild conditions, directly and smartly, in only one step. 

### 3.1. Intermolecular Aza-Piancatelli Rearrangement

**Scheme 15:**
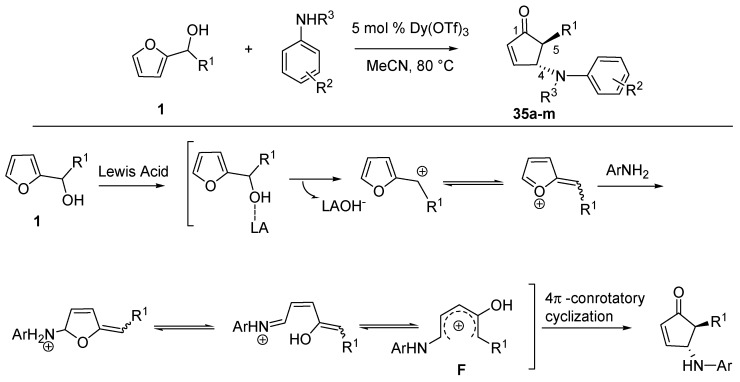
Aza-Piancatelli rearrangement and a proposed reaction mechanism.

Recently Read de Alaniz’s group employed 2-furylcarbinols and a series of anilines to access *trans*-4-amino-5-substituted-cyclopent-2-enones (**35**), appealing structures for the synthesis of biologically active compounds [44] (Scheme 15).

This was allowed thanks to the identification of catalysts such as lanthanoid salts which were able to selectively activate 2-furaldehydes in the presence of an excess of nucleophilic amines [45]. Under optimized conditions, the aza-version of the Piancatelli rearrangement was carried out in acetonitrile at 80 °C, together with a catalytic amount (5 mol %) of Dy(OTf)_3_ [46], preferred over Sc(OTf)_3_ which gave similar results, but it is more expensive. 

The mechanism is proposed to involve the elimination of the hydroxy-group through coordination and activation by the Lewis acid. The resulting furylcation undergoes nucleophilic attack by the aniline at the 5 position of the ring, thus starting the cascade reaction that forms the product (Scheme 15). The 1-amino-4-hydroxy pentadienyl cation **F** is analogous to **B** (Scheme 2) and it is supposed to undergo the 4π-conrotatory electrocyclization, thus explaining the high *trans*-diasteroselectivity of the reaction.

The synthesized products (Table 1, **35a**–**m**) were isolated as single diastereomers and they demonstrate that the rearrangement is compatible with several substituents. Yields ranging from 62% to 93% were obtained with different anilines in combination with 2-furylcarbinols (**1**) bearing R^1^ as a simple phenyl (**35a**–**c**), a substituted aryl possessing an electron-donating (**35d**–**e**) or an electron-withdrawing group (**35f**–**g**). Furthermore, the presence of an aliphatic group as R^1^ (**35h**–**k**) was also tolerated and the reaction worked well with both primary and secondary anilines (**35l**–**m**).

**Table 1:** Aza-Piancatelli rearrangement products and yields.

Compound	R^1^	R^2^	R^3^	Yield (%) *
35a	Ph	H	H	86
35b	Ph	4-I	H	93
35c	Ph	4-MeO	H	62
35d	4-MeOC_6_H_4_	4-I	H	68
35e	4-MeOC_6_H_4_	2,4,6-triMe	H	89
35f	4-CF_3_C_6_H_4_	4-I	H	83
35g	4-CF_3_C_6_H_4_	2,4,6-triMe	H	78
35h	Me	4-I	H	68
35i	Me	2,4,6-triMe	H	74
35j	*i-*Pr	4-I	H	73
35k	*i-*Pr	2,4,6-triMe	H	89
35l	Ph	H	CH_3_	74
35m	Ph	4-Br	CH_3_	88

* Conditions: 5% mol Dy(OTf)_3_, MeCN, 80 °C.

**Scheme 16:**
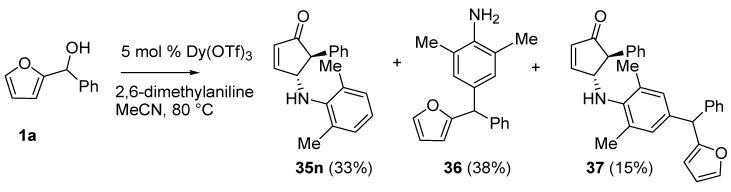
Rearrangement with 2,6-dimethylaniline.

No isomerization products (4-amino-2-substituted-cyclopent-2-enones) were found using the aza-Piancatelli rearrangement because of the mild reaction conditions [46]. The reaction was successful with sterically hindered 2,4,6-trimethylaniline (35e, 35g, 35i, 35k), while a competitive Friedel-Craft alkylation occurred when 2,6-dimethylaniline was employed delivering **36** and **37** (Scheme 16).

**Scheme 17:**
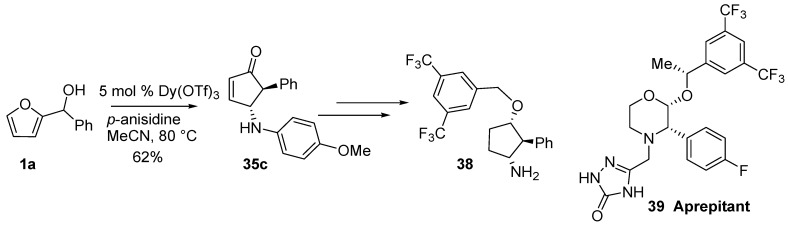
Application to the synthesis of an hNK1 inhibitor.

Compound **35c** (Table 1) was converted into racemic **38** which represents a key intermediate for the preparation of a mimetic of the morpholinic core of the hNK1 antagonist Aprepitant (**39**) (Scheme 17) [47,48]. 

Subba Reddy *et al.* [49] performed the aza-Piancatelli rearrangement using phosphomolybdic acid (PMA) as the catalyst (0.03 mol %) in acetonitrile at reflux, isolating *trans*-4-amino-5-substituted cyclopent-2-enones in greater than 80% yield, in about 1 h.

**Scheme 18:**
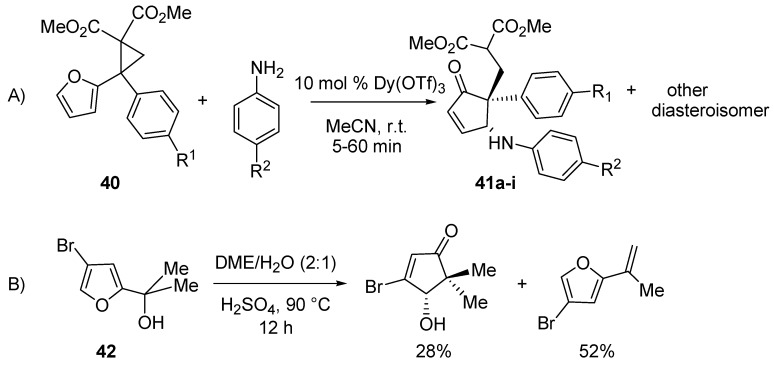
Rearrangement of differently functionalized furans.

A further advance in the aza-Piancatelli rearrangement has been recently published and allowed the synthesis of cyclopent-2-enones with a quaternary carbon atom at the 5 position in high diasteroselectivity [50]. Taking advantage of the reactivity of polarized donor-acceptor (D-A) cyclopropanes (**40**) [51,52] due to ring strain [53] and their behavior as carbocation upon Lewis acid activation, the Read de Alaniz group found an alternative method to trigger the rearrangement and obtain highly functionalized cyclopent-2-enones (**41**) (Scheme 18, A). The protocol obviated the problems that Piancatelli and D’Auria [15] encountered when they tried to prepare cyclopent-2-enones with a quaternary carbon through the Piancatelli rearrangement starting from a furan with a tertiary carbinol side chain (**42**) and a large amount of dehydrated compound was found together with the rearranged product (Scheme 18, B).

The best results in terms of yields (57%–89%), diasteroselectivity (6:1 up to 60:1) and reaction times (5–60 min) were found with 10 mol % Dy(OTf)_3_ in acetonitrile at room temperature. The influence of R^1^ and R^2^ was investigated (Table 2).

*Para*-substituted anilines with electron-withdrawing groups led to higher diastereoselectivities (**41c** *vs.* **41a**, **41i** *vs.* **41h**) than the corresponding anilines bearing electron-donating groups (**41b** *vs.* **41a**, **41g** *vs.* **41h**). But when R^1^ was a methoxy moiety, good yields and diasteroselectivities (**41d**–**f**) were observed with anilines possessing either an electron-donating or electron-withdrawing group.

**Table 2:** Rearrangement with (D–A) cyclopropanes.

Compound	R^1^	R^2^	Yield (%) *	dr
41a	H	H	89	13:1
41b	H	MeO	57	6:1
41c	H	CF_3_	72	60:1
41d	4-MeO	MeO	87	25:1
41e	4-MeO	H	84	32:1
41f	4-MeO	CF_3_	63	22:1
41g	4-CN	MeO	58	2:1
41h	4-CN	H	76	1:1
41i	4-CN	CF_3_	65	5:1

* Conditions: 10 mol % Dy(OTf)_3_, MeCN, r.t.

When R^1^ was an electron-withdrawing group (e.g., CN, CF_3_), diasteroselectivities were poor (**41g**–**i**) and the resulting products were less stable. In fact an intramolecular Michael addition frequently occurred, even during column chromatography, delivering bicyclic compounds that could not be isolated from the reaction mixture. With R^1^ = R^2^ = CF_3_, compound **43** was obtained in high yield (83%)_,_ by forcing this side-reaction under basic conditions.

**Figure 2:**
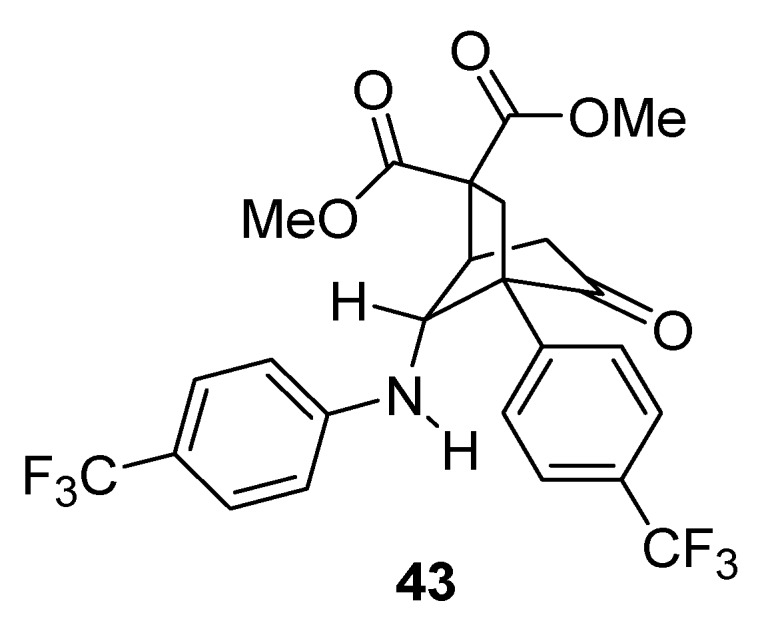
Michael addition side-product.

The X-ray crystal structure analysis demonstrated that only the major diastereomer deriving from the rearrangement underwent cyclization [50] (Figure 2).

Diastereoselectivity was observed to be dependent on the reaction temperature. In fact, when the rearrangement was carried out at 80 °C, the diastereomeric ratio improved to 30:1 for **41a**, to 16:1 for **41b** and to 22:1 for **41i**.

### 3.2. Intramolecular Aza-Piancatelli Rearrangement

In 2011 Read de Alaniz published the first example of an intramolecular version of the aza-Piancatelli rearrangement [54]. This approach was based upon Piancatelli’s observation that suitable 5-substituted-2-furylcarbinols could rearrange under certain conditions [14] and upon the protocol of Yin on the synthesis of oxabicyclic cyclopentenones (**6**) starting from 2-furylcarbinols (**3**) bearing a hydroxyalkyl side chain at the 5 position of the furan ring (Scheme 5) [8,11]. 

**Scheme 19:**
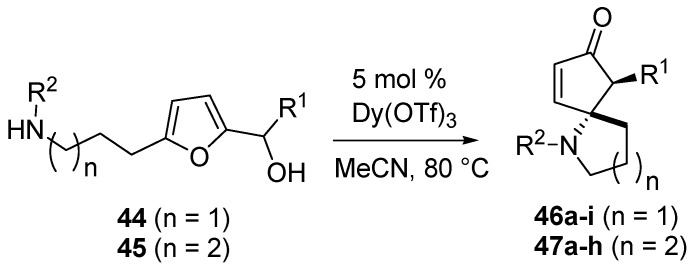
Intramolecular aza-Piancatelli rearrangemet and synthesis of azaspirocycles.

The authors worked with 2-furylcarbinols bearing an aminoalkyl chain at the 5 position of the furan ring (**44**–**45**) and generated azaspirocyclic scaffolds (**46**–**47**, Scheme 19) [54]. This densely functionalized framework was obtained in only one step, as a single diastereomer, with high efficiency and in high yield, differently from the known procedures that require several synthetic steps for the construction of the tertiary carbon center bearing the nitrogen atom, and the formation of the spirocyclic ring [55,56].

The optimized conditions for the intermolecular rearrangement (Dy(OTf)_3_ 5 mol % in refluxing acetonitrile) [44] turned out to be suitable also for the intramolecular version, and the mechanism previously hypothesized was also assumed to be in action (Scheme 15). As shown in Table 3 for the formation of 5-azaspirocycles **46** (*n* = 1), the rearrangement worked equally well for most common substituents, but the nature of R^2^ had a significant impact on the outcome of the reaction, both in terms of yield and reaction rate. 

**Table 3:** 5-Azaspirocycles (**46**, *n* = 1) *via* aza-Piancatelli rearrangement.

Compound	R ^1^	R ^2^	Time	Yield (%) *
46a	Ph	Ph	15 min	96
46b	Ph	4-MeOC_6_H_4_	150 min	74
46c	4*-*NO_2_C_6_H_4_	Ph	5 h	67
46d	4*-*BrC_6_H_4_	Ph	5 h	84
46e	H	Ph	15 h	90
46f	H	4*-*MeOC_6_H_4_	48 h	57 ^#^
46g	4-MeOC_6_H_4_	4-CF_3_C_6_H_4_	15 min	78
46h	4-MeOC_6_H_4_	4-IC_6_H_4_	5 min	84
46i	CH_3_	Ph	15 min	91

* Conditions: 5 mol % Dy(OTf)_3_, MeCN, 80 °C; ^#^ 10 mol % cat. Required.

When R^2^ was an electron-rich group, as in the case of PMP-cleavable *N*-protecting group, yields were lower and longer reaction times were required (**46a** *vs.* **46b**, **46e** *vs.* **46f**), while the presence of CF_3_- or I- on the aryl at R^2^ led to faster rearrangement (**46g** and **46h**). NO_2_- or Br-substituted aryl groups at R^1^ increased the reaction times (**46c** and **46d**). Furthermore, a methyl group at R^1^ was very well tolerated (**46i**). A similar effect of the R1 and R2 substitution occurred in the synthesis of 6-azaspirocycles 47 (n = 2). Electron-rich anilines required longer reaction times than unsubstituted ones (**47a** *vs.* **47b**; **47d** *vs.* **47f**, Table 4), and in the case of **47f** a 20 mol % catalyst was required to achieve a modest yield. On the contrary, the presence of a halogen had a beneficial effect on the reaction rate (**47c**, **47e** and **47h**).

**Table 4:** 6-Azaspirocycles (47, *n* = 2) via intramolecular aza-Piancatelli rearrangement.

Compound	R ^1^	R ^2^	Time	Yield (%) *
47a	Ph	Ph	1 h	75
47b	Ph	4-MeOC_6_H_4_	75 h	74
47c	Ph	4-IC_6_H_4_	5 min	69
47d	H	Ph	48 h	70
47e	H	4-IC_6_H_4_	8 h	90
47f	H	4-MeOC_6_H_4_	72 h	37 ^#^
47g	nBu	Ph	15 h	54
47h	nBu	4-IC_6_H_4_	2 h	65

* Conditions: 5 mol % Dy(OTf)_3_, MeCN, 80 °C. ^#^ 20 mol % cat. Required.

### 3.3. Intramolecular Oxa-Piancatelli Rearrangement

**Scheme 20:**
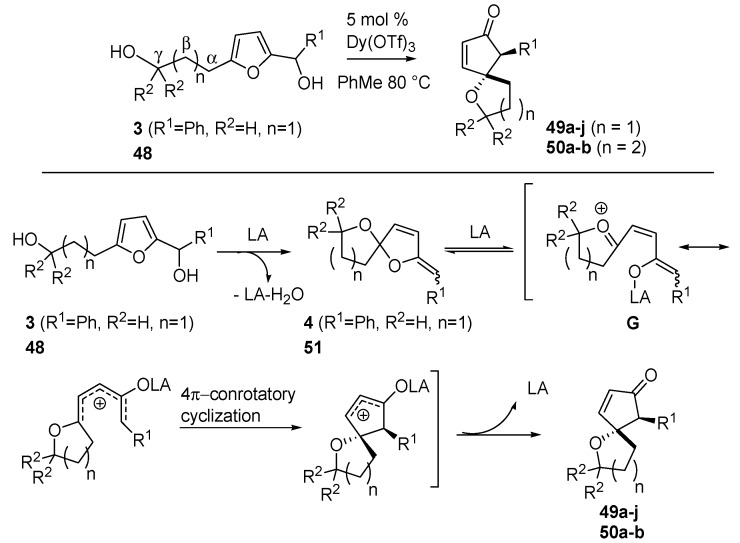
Intramolecular oxa-Piancatelli rearrangement and proposed mechanism

Read de Alaniz also explored the oxa-Piancatelli version of the rearrangement on substrates bearing a hydroxy-alkyl moiety at the 5 position of the furan ring (**3**, see Scheme 5, and **48**) [57] and thereby accessing oxaspirocycles (**49** and **50**) (Scheme 20) in only one step and in a highly diasteroselective manner, thus avoiding the usually performed multistep procedures [58,59,60].

According to Scheme 20, 2-furylcarbinols **3** and **48** lead to the formation of spiroketal enol ether **4** and **51** upon acidic catalysis. The coordination of Dy(OTf)_3_ delivers intermediate **G** which, according to the canonical 4π-conrotatory cyclization, affords spirocyclic ethers **49** and **50**. The optimized conditions required 5 mol % Dy(OTf)_3_ in toluene at 80 °C. It has to be mentioned that the use of refluxing acetonitrile, the best solvent for the aza-Piancatelli rearrangement, in this case led to decomposition. 

In most cases the products were isolated in reasonable to excellent yields and with the expected high *trans*-selectivity (Table 5). The reaction proceeded efficiently (compounds **49a**–**c**), but in the case of strong electron-withdrawing groups as R^1^, harsher conditions (higher temperature and longer reaction time) were required in respect to the aza-rearrangement (compare **49d** and **46c**, Table 3). The presence of a heterocycle was also tolerated (**49e**).

**Table 5:** Oxaspirocycles (**43** and **47**) via oxa-Piancatelli rearrangement.

Compound	R^1^	R^2^	n	Time	Yields (%) *
49a	Ph	H	1	6 h	91
49b	4-MeOC_6_H_4_	H	1	20 h	89
49c	4-BrC_6_H_4_	H	1	24 h	83
49d	4-NO_2_C_6_H_4_	H	1	7 days	75 ^#^
49e	2-thiophene	H	1	4 h	90
49f	Ph	CH_3_	1	2 h	74
49g	*i-*Pr	H	1	-	0
49h	*i-*Pr	CH_3_	1	1 h	25
49i	*n-*Bu	H	1	-	0
49j	*n-*Bu	CH_3_	1	2 h	98
50a	Ph	H	2	-	0
50b	Ph	CH_3_	2	16 h	20

^*^ Conditions: 5 mol % Dy(OTf)_3_, toluene, 80 °C. ^#^ 16 h, 100 °C.

In contrast to the aza-Piancatelli reaction, aliphatic R^1^ groups delivered complex reaction mixtures (**49g** and **49i**), except in the case where a *gem*-dimethyl moiety was present in position γ (Scheme 20). The beneficial effect of this substitution is not clear, but the Authors hypothesize a more favoured formation of intermediate **G** because the *gem*-dimethyl group prevents Lewis acid from coordinating the vicinal oxygen atom. The *gem*-dimethyl effect is pronounced also in the formation of the 6-oxaspirocycles (**50a** and **50b**).

Further investigations of the effects of substituents along the aliphatic chain (α, β and γ positions, Scheme 20) were carried out revealing that only groups in the γ or β were tolerated, while the presence of a α substitution hampered the rearrangement.

### 3.4. Domino Aza-Piancatelli/Hetero-Michael Reaction

**Scheme 21:**
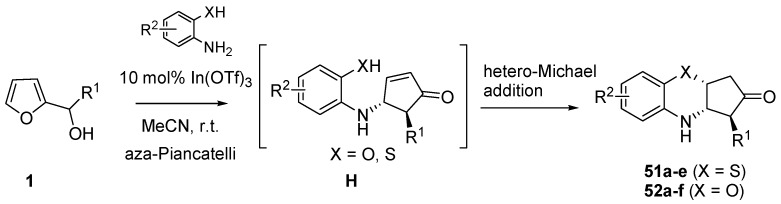
Domino aza-Piancatelli/hetero-Michael addition products.

Recently Subba Reddy’s group illustrated a further application of the aza-Piancatelli reaction by a new route to 3,4-dihydro-2H-benzo[*b*][1,4]oxazine and thiazine derivatives, core structures shared among several biologically and pharmacologically active compounds [61]. This new procedure is based on a domino aza-Piancatelli/hetero-Michael addition conducted by reacting 2-furylcarbinols with 2-aminophenols or 2-aminothiophenols (Scheme 21). 

Preliminary experiments with 10% phosphomolybdic acid gave products in rather good yields (75%, 2.5 h), but the use of indium salts as catalysts [62,63,64,65] (10 mol% In(OTf)_3_) in acetonitrile at room temperature allowed the optimization of the synthesis of 3,4-dihydro-2H-benzo[*b*][1,4] thiazine derivative (**51a**) up to 86% in terms of yields, with high diastereoselectivity and shorter reaction times (2 h) [61].

The initial steps of the mechanism are the same as proposed for the aza-Piancatelli (see Scheme 15). Then after the 4π-conrotatory cyclization which leads to intermediate **H**, a thia/oxa-conjugate addition finally gives compounds **51** or **52**, that show a *trans* relationship between R^1^ and N, resulting from the aza-Piancatelli rearrangement, and a *cis* orientation between N and X (O or S) arising from the conjugate addition (Scheme 21).

In Table 6, some compounds obtained with the domino approach are illustrated. The isolated yields were high both with 2-aminothiophenols and 2-aminophenols, although 2-aminophenols required slightly longer reaction times, being less reactive.

**Table 6:** Derivatives obtained via domino aza-Piancatelli/hetero-Michael reaction with In(OTf)_3_.

Compound	R^1^	R^2^	X	Time (h)	Yields (%) *
51a	Ph	H	S	2.0	86
52a	Ph	5-Me	O	3.5	73
51b	Ph	4-Cl	S	2.5	85
52b	Ph	4-Cl	O	3.5	78
51c	4-MeOC_6_H_4_	H	S	2.0	87
52c	4-MeOC_6_H_4_	H	O	3.0	75
51d	4-MeOC_6_H_4_	4-Cl	S	2.5	88
52d	4-MeOC_6_H_4_	4-Cl	O	2.5	82
51e	4-FC_6_H_4_	H	S	2.5	85
52e	4-FC_6_H_4_	H	O	3.5	81
52f	4-FC_6_H_4_	4-Cl	O	3.5	80

* Conditions: 10 mol % In(OTf)_3_, MeCN, r.t.

Curiously, nearly two months later, another group published [66] the same reaction sequence shown in Scheme 21 using La(OTf)_3_ (5 mol %) as the catalyst and acetonitrile at reflux as the solvent. For the same substrates, yields were lower if compared with those obtained with In(OTf)_3_ and reaction times were longer (Table 7). Moreover 2-aminothiophenols failed to react under these conditions.

**Table 7:** Oxazines obtained via domino aza-Piancatelli/hetero-Michael reaction with La(OTf)_3_.

Compound	R^1^	R^2^	Yields (%)^*^
52g	Ph	H	81
52b	Ph	4-Cl	66
52c	4-MeOC_6_H_4_	H	46
52d	4-MeOC_6_H_4_	4-Cl	51
52e	4-FC_6_H_4_	H	46
52f	4-FC_6_H_4_	4-Cl	78

* Conditions: 5 mol % La(OTf)_3_, MeCN, 80 °C, 4 h.

**Scheme 22:**
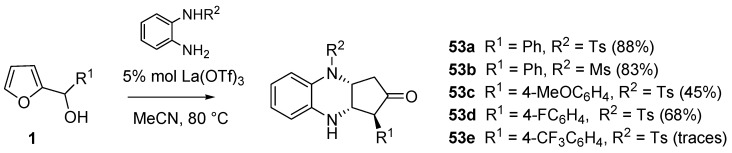
Diazines (**53**) via domino aza-Piancatelli/hetero-Michael addition.

Complex mixtures or no reaction were observed in the attempts of building [1,4]heterocycles different from benzoxazine, starting from building blocks such as *o*-phenylenediamines, pyrocatechols or 2-aminopyridin-3-ol. The introduction of an electron-withdrawing group (e.g., methanesulfonyl, tosyl) on one of the nitrogens of *o*-phenylenediamines proved beneficial for the isolation of the desired products (Scheme 22, **53a**–**d**). On the other hand, despite the electron-withdrawing group in R^2^, the presence of an electron-poor moiety in R^1^ hampered the reaction to occur (**53e**).

### 3.5. Intramolecular C-Piancatelli Rearrangements

**Scheme 23:**
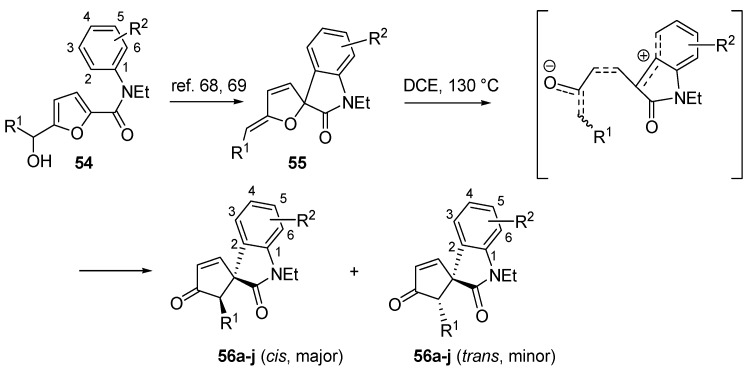
Synthesis of spirocyclopentenoneoxindoles (**56**) from 2-furylcarbinols (**54**).

A very recent variant of this transformation, which is also the first example of an intramolecular C-Piancatelli rearrangement, has been reported by Yin and co-workers [67]. When treating 2-furylcarbinols functionalized with an electron-rich aromatic tertiary amide (**54**) in the presence of acidic catalysts, a nucleophilic Friedel-Craft attack from the *ortho* position onto the oxa-carbenium intermediate occurs, forming the spirofurooxindole derivative (**55**) (Scheme 23) [68,69]. Since these compounds are structurally similar to intermediate **A** (Scheme 2) in the proposed Piancatelli rearrangement mechanism, the authors envisioned the possibility to promote the rearrangement through ring opening and cyclization under suitable reaction conditions, thus accessing the novel core scaffold spirocyclopenten-2-oneoxindoles (**56**) as a mixture of *cis*/*trans* isomers [67]. The use of a catalyst (Pd^II^ complexes or Lewis acids) led to a mere 40% yield, while a simple solvolysis without the use of a catalyst proved to be more successful. The use of 1,2-dichloroethane (DCE) at 130 °C increased yields over 80%.

The influence of substituents R^1^ and R^2^ on diasteroselectivity is highlighted in Table 8. The lack of a methoxy group at the 3-position of R^2^ leads to a lower diasteroselectivity (**56a**–**b** *vs.* **56c**, **56e** *vs.* **56f**, **56g** *vs.* **56h**), while an increase is seen if an *ortho*-substituent is present at R^1^ (**56d** *vs.* **56g**). 3,4,5-Trimethoxy derivatives combined with an *o*-substituent enabled almost exclusively *cis*-isomer isolation (**56e**, **56g** and **56i**).

**Table 8:** Synthesis of spirocyclopenten-2-oneoxindoles.

Compound	R ^1^	R ^2^	*cis/trans*	Yield (%) *
56a	Ph	3,4,5-tri-MeO	4/1	94
56b	Ph	3,5-di-MeO	5/1	91
56c	Ph	4,5-di-MeO	1.5/1	89
56d	4-ClC_6_H_4_	3,4,5-tri-MeO	5.6/1	93
56e	2-FC_6_H_4_	3,4,5-tri-MeO	>99/1	87
56f	2-FC_6_H_4_	4,5-di-MeO	3/2	85
56g	2-ClC_6_H_4_	3,4,5-tri-MeO	>99/1	92
56h	2-ClC_6_H_4_	4,5-di-MeO	1.2/1	89
56i	2-MeC_6_H_4_	3,4,5-tri-MeO	>99/1	94
56j	2-MeC_6_H_4_	3,5-di-MeO	5/1	91

* Conditions: DCE, 130 °C.

## 4. Conclusions

Since its discovery in 1976, the Piancatelli rearrangement has appeared as a versatile reaction for the construction of substituted cyclopent-2-enones convenient for the synthesis of prostaglandin derivatives. Several groups have been investigating new applications of the reaction that culminated in the recent publication of a number of fascinating papers. The use of alternative nucleophiles to water in both inter- and intramolecular reactions has allowed access to several attractive and complex chemotypes, such as azaspirocycles and spirocyclic ethers. Moreover, the combination with a subsequent intramolecular conjugate addition permits the synthesis of thiazines and oxazines in a straightforward manner. These are promising scaffolds for the synthesis of complex natural products and biologically active compounds.

In summary, the Piancatelli rearrangement turned out to be an elegant approach for the stereoselective construction of complex scaffolds that traditional methods would generate *via* multistep processes and with limited diversity on the substituents. Thanks to the use of milder reaction conditions, this recently re-discovered rearrangement holds promises for a number of new applications. An enantioselective version would represent a further progress in the field, for instance in the synthesis of oxaspirocycles with the use of chiral Lewis acids.
